# Supplementary material for: Sequence dependence of transient Hoogsteen base pairing in DNA
Source: PLoS Comput Biol. 2022 May 26;18(5):e1010113. doi: 10.1371/journal.pcbi.1010113 (PMC9177043; doi:10.1371/journal.pcbi.1010113)
Supplement: S1 Text — (PDF) [file pcbi.1010113.s001.pdf]

S1 Text for
Sequence dependence of transient Hoogsteen base pairing in DNA

Alberto Pérez de Alba Ortíz[1,2], Jocelyne Vreede[1], Bernd Ensing[1,3*],

1 Van 't Hoff Institute for Molecular Sciences and Amsterdam Center for Multiscale Modeling, University of Amsterdam, Amsterdam, The Netherlands.
2 Soft Condensed Matter, Debye Institute for Nanomaterials Science, Utrecht University, Utrecht, The Netherlands.
3 AI4Science Laboratory, University of Amsterdam, Amsterdam, The Netherlands

* b.ensing@uva.nl

Adjustments to the general simulation protocol

For the outside path of sequence GAA, we average the metadynamics free-energy estimation from 1 to 6 ns. For the outside path of sequence CAG, we average the metadynamics free-energy estimation from 4 to 9 ns. For the outside path of sequence CAC, we average the metadynamics free-energy estimation from 1 to 4 ns. For both paths of sequence TAA and for the inside path of sequence CAT, we decrease the half-life to 10 ps, the attractor restraint force constant to 50 kcal/mol and the tube potential force constant to 20 kcal/mol. These changes are done either because the simulations failed to converge before the default time window (2 to 7 ns), or because they did not optimize to the desired pathway within the default time window with the general half-life and restraint parameters.

Collective variables

The collective variables (CVs) used in this work to bias the Watson-Crick-Franklin (WCF) to Hoogsteen (HG) base-pairing transition of the A16·T pair are:

- χ': The A16 base rolling torsion around the glycosidic bond defined by the pseudo-dihedral angle formed by the axis C1'-N9 and the vectors P2-P1 and P5-P6. Taken from [2] and shown in Fig C.
- θ: The A16 base flipping torsion defined by the pseudo-dihedral angle (P1+P2)-P3-P4-P5. Taken from [3] and shown in Fig C.

Additionally, we analyze the following CVs describing different structural features of DNA, taken from [2]:

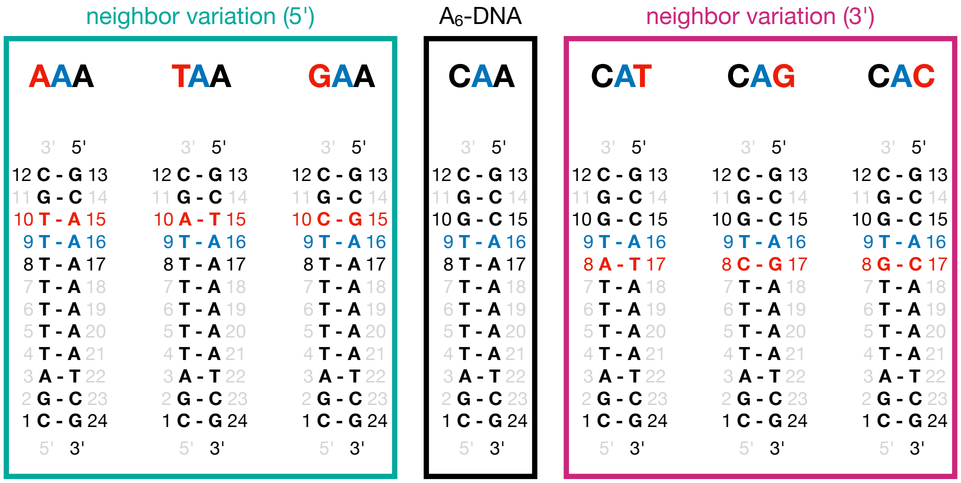

Fig A. DNA sequence variations for which the transition from WCF to HG base pairing of A16·T9 is studied. The transitioning base pair A16·T9 is shown in blue. The original sequence A_6-DNA from [1] is outlined in black, and has the local environment CAA. Variations of the direct neighbor of A16 in the 5' direction are outlined in teal, and have the local environments AAA, TAA and GAA. Variations of the direct neighbor of A16 in the 3' direction are outlined in magenta, and have the local environments CAT, CAG and CAC.

- d_{WCF}: The distance of the characteristic WCF H-bond between A16 (N1) and T9 (N3), shown in Fig C.
- d_{HG}: The distance of the characteristic HG H-bond between A16 (N7) and T9 (N3), shown in Fig C.
- d_{HB}: The distance of the conserved H-bond, present both in WCF and in HG, between A16 (N6) and T9 (O4), shown in Fig C.
- d_{CC}: The distance between A16 (C1') and T9 (C1'), shown in Fig C.
- d_{NB}: The distance between the neighboring bases of A16, described by the centers of mass P1' and P2', shown in Fig C.

Table A. Stable-state characterization based on averages and standard deviations of several CVs during equilibrations at the WCF and the HG states. The sequences are as depicted in Fig A. The CVs—defined in [2]—describe the following structural changes. χ': A16 rolling angle; θ: A16 flipping angle; d_{WCF}: distance of the characteristic WCF hydrogen-bond; d_{HG}: distance of the characteristic HG hydrogen-bond; d_{HB}: distance of the conserved hydrogen-bond in both states; d_{CC}: distance between the backbone C1' atoms of A16 and T9; d_{NB}: distance between the neighboring nucleotides of A16.

CV	state	AAA	TAA	GAA	CAA	CAT	CAG	CAC
χ'	WCF	1.6±0.17	1.5±0.18	1.6±0.17	1.5±0.15	1.5±0.15	1.4±0.15	1.4±0.15
(rad)	HG	-1.5±0.19	-1.8±0.19	-1.6±0.18	-1.7±0.17	-1.7±0.17	-1.7±0.17	-1.8±0.17
θ	WCF	-0.1±0.09	-0.1±0.12	-0.1±0.08	-0.1±0.11	-0.1±0.09	-0.1±0.11	-0.1±0.09
(rad)	HG	0.0±0.05	-0.0±0.08	-0.0±0.06	-0.0±0.05	-0.0±0.05	-0.0±0.06	-0.0±0.06
d_{WCF}	WCF	3.0±0.12	3.0±0.12	3.0±0.13	3.0±0.12	3.0±0.12	3.0±0.13	3.0±0.13
(Å)	HG	5.8±0.21	6.0±0.58	6.0±0.48	5.9±0.19	6.0±0.24	6.0±0.28	6.0±0.32
d_{HG}	WCF	6.4±0.15	6.4±0.14	6.4±0.15	6.4±0.14	6.4±0.14	6.4±0.15	6.5±0.15
(Å)	HG	3.1±0.2	3.3±0.66	3.2±0.55	3.2±0.49	3.1±0.35	3.1±0.43	3.2±0.57
d_{HB}	WCF	3.0±0.18	3.0±0.2	3.0±0.19	3.0±0.17	3.0±0.2	3.0±0.2	2.9±0.16
(Å)	HG	2.9±0.22	3.0±0.71	3.0±0.52	3.0±0.42	2.9±0.16	2.9±0.18	2.9±0.23
d_{CC}	WCF	10.6±0.28	10.6±0.29	10.7±0.29	10.6±0.29	10.6±0.28	10.7±0.31	10.8±0.3
(Å)	HG	9.0±0.33	9.2±0.6	9.2±0.64	9.0±0.35	9.1±0.56	9.1±0.69	9.3±0.83
d_{NB}	WCF	7.5±0.31	7.9±0.4	7.4±0.3	7.9±0.36	7.6±0.44	7.8±0.41	7.8±0.41
(Å)	HG	7.6±0.31	7.9±0.4	7.4±0.29	7.8±0.38	7.7±0.36	7.9±0.4	8.0±0.4

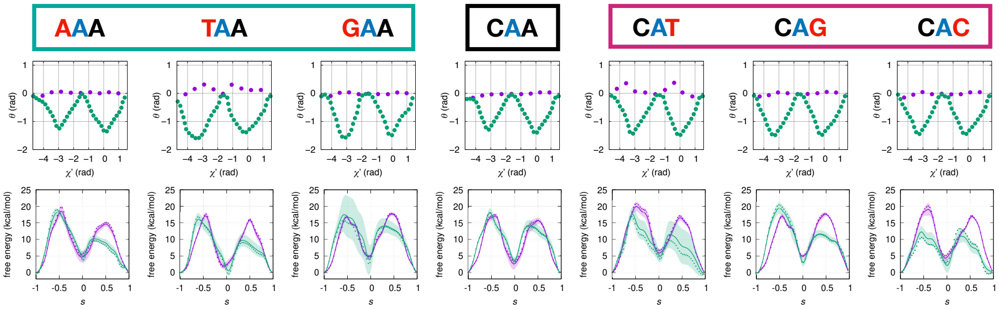

Fig B. Transition pathways (top) and free-energy profiles (bottom) for the inside and outside mechanisms of WCF-to-HG base-pairing with local sequence variations. Sequences are as shown in Fig A. Variations of the direct neighbor of A16 in the 5' direction are outlined in teal, while in the 3' direction they are outlined in magenta. Inside paths are depicted in purple and outside paths in green. The free-energy profiles and their error bars are depicted by the solid lines and shadowed regions. The dotted lines show the free-energy profiles upon reducing the sampling time by 2 ns. Sampling follows the scheme presented in Fig 1E in the main text.

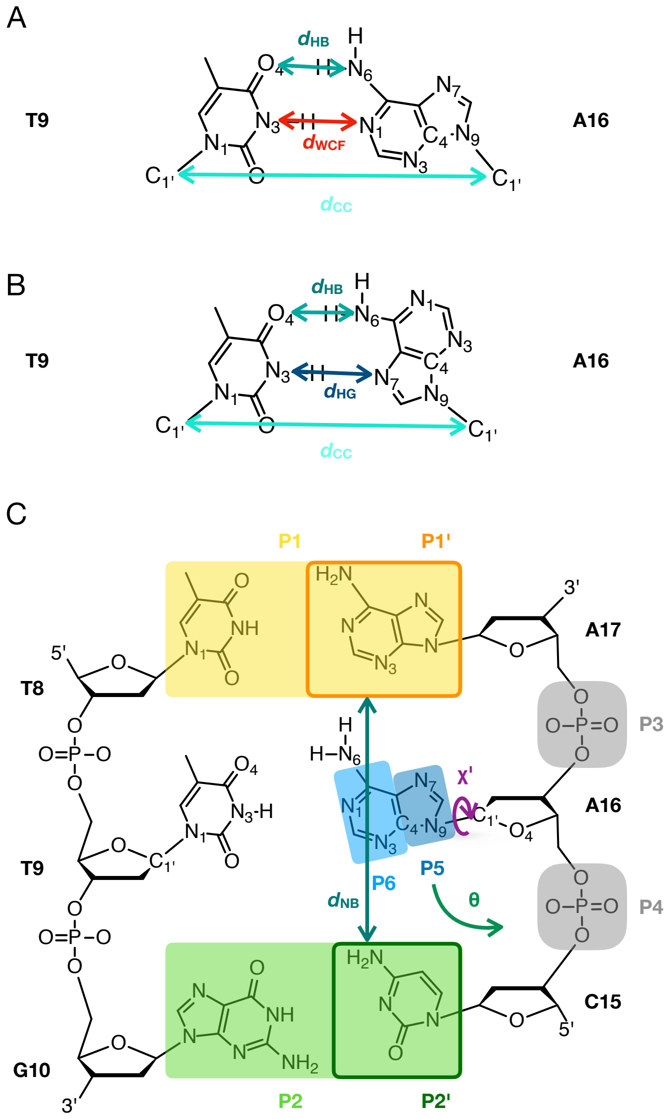

Fig C. Scheme of the CVs biased or analyzed during the WCF-to-HG base-pairing transition. A: WCF base pair with graphical representations of the CVs: d_{WCF}, d_{HB} and d_{CC}. and HG base pair with graphical representations of the CVs: d_{HG}, d_{HB} and d_{CC} (bottom); **B**: HG base pair with graphical representations of the CVs: d_{HG}, d_{HB} and d_{CC} (bottom). **C**: A16·T9 bp and its two neighboring bps with graphical representations of the centers of mass involved in the calculation of CVs: χ', θ and d_{NB};

References

1. Nikolova EN, Kim E, Wise AA, O'Brien PJ, Andricioaei I, Al-Hashimi HM. Transient Hoogsteen base pairs in canonical duplex DNA. Nature. 2011;470(7335):498–502.

2. Pérez de Alba Ortíz A, Vreede J, Ensing B. The adaptive path collective variable: a versatile biasing approach to compute the average transition path and free energy of molecular transitions. In: Bonomi M, Camilloni C, editors. Biomolecular Simulation. Springer; 2019. p. 255–290.

3. Song K, Campbell AJ, Bergonzo C, de los Santos C, Grollman AP, Simmerling C. An improved reaction coordinate for nucleic acid base flipping studies. Journal of chemical theory and computation. 2009;5(11):3105–3113.

.
