# Supplementary material for: Sequence dependence of transient Hoogsteen base pairing in DNA
Source: PLoS Comput Biol. 2022 May 26;18(5):e1010113. doi: 10.1371/journal.pcbi.1010113 (PMC9177043; doi:10.1371/journal.pcbi.1010113)
Supplement: Attachment — Submitted filename: plos_reply.pdf [file pcbi.1010113.s002.pdf]

FACULTY OF SCIENCE
van 't Hoff Institute for Molecular Sciences

Response to Reviewers

of "Sequence dependence of transient Hoogsteen base pairing in DNA"
by A. Pérez de Alba Ortiz, J. Vreede and B. Ensing

PLOS Computational Biology

March 18th, 2022

We sincerely thank the reviewers for their valuable and constructive remarks. Below, we list point-by-point the queries and comments of the three reviewers (in black font), together with our response and a description of the changes made to the manuscript (in blue font). Changes in the revised manuscript are also indicated in blue font.

Reviewer #1: Ensing and co-workers report on the pathways and free energies of Watson-Crick to Hoogsteen base pairing in A6 DNA with multiple sequence contexts in MultiPMD (multiple-path-metadynamics) simulations exploring two collective variables that enable the conversion. Overall the manuscript is well written and well summarizes the findings from the simulations. Of some concern is the short sampling time (< 10 ns per walker), results from single (sets of) simulations , and potential bias of attracters, repellers, and force constants on the results. For example, if simulations are run twice as long per walker or with twice as many walkers are consistent results obtained? In the conclusion, it is stated you need much less simulation time that the microsecond timescales seen previously – to better justify, perhaps present results that show convergence – for example, do independent runs on same sequence (with different arrangement of ions or initial velocities) give equivalent results? Figure S2 suggests poor convergence (or high variance) in the GAA and CAT sequences.

We thank Reviewer #1 for an attentive analysis and valuable comments. Indeed, the sampling times are orders of magnitude shorter that those of previous works. There are two main reasons for this incremented efficiency. First is the fact that, as pointed out by Reviewer #1, we employ multiple walkers. Such schemes have been demonstrated to decrease the required sampling time linearly with the number of walkers [30]. Second, and most impactful, is that the path-based approach allows us to focus the sampling on the transition channel, rather than on the entire (χ',θ)-plane. This approach reduces the sampling time exponentially with respect to the number of collective variables involved in the calculation [22,29]. Moreover, since the attractors already localize the paths for the inside and outside mechanisms, the time spent finding the transition channel is also shortened. We had already performed a similar test to the one suggested by Reviewer #1, but kept only the result corresponding to the longer sampling time. In Fig. S2, we have added dotted lines showing that the free-energy profiles are mostly unchanged if the sampling time is decreased by 2 ns.

The increased error bars for sequences GAA and CAT are due to the relatively high variance, or width, of the mechanistic outside pathway. For all sequences, the inside and outside routes marked by the attractors lie within a continuous spectrum of pathways, although for most sequences the intermediate pathways are less relevant (i.e. have a higher barrier region) than our selected inside and outside routes. For GAA and CAT, we observe a higher variance due to this branching of the inside and outside pathways, which overlap at the stable states. This could perhaps be improved by further tuning the tube potential and the attractor position, but that would hamper direct comparison between the different sequences. Moreover, we find it interesting to observe the larger variance in the outside paths and later branching between the inside and outside paths, which we now explain in the main text

Changes to the text:

- We have added dotted lines to Fig. S2 in the Supporting Information; showing the change of the free-energy results after subtracting 2 ns of sampling. We point this out also in Results and Discussion.

- We have added an explanation about the error bars for the outside pathway in sequences GAA and CAT in Results and Discussion.
- We added reference [30] for multiple walkers.

Minor points

- What ion parameters?
- (see Fig. S1, i.e. – needs closing ")"

We thank Reviewer #1 for these additional comments. The ion parameters are taken from the AMBER99 force field.

Changes to the text:

- We added the ion parameters and the corresponding reference in Materials and methods.
- The missing closing parenthesis has also been corrected in Results and Discussion.

Reviewer #2: Perez et al. reported the in- and out-HG pairing pathways using the adaptive path sampling method. Also, they investigated sequence dependent effects on those pathways.
In this computational study, they concluded that the out-path is more favorable than in-path. This is interesting. Overall, this manuscript was well written and the results are well presented.
I recommend this manuscript be published in the PLOS comp. Bio. after some minor revisions:

1. a.The authors need to describe in detail how the free energy profile along the paths were obtained. b.I am wondering if these free energy profiles on their optimized paths be exactly reproduced by more conventional US-WHAM or other non-equilibrium schemes along those paths? If they are, please give a proper reference on this.

We thank Reviewer #2 for the valuable comments. The free-energy profiles along the path progress parameter, s, and their error bars are obtained by time-averaging metadynamics-based estimations. Our protocol is as follows:

1. We run metadynamics for at least 7 ns.
2. We verify when the dynamics achieve the "free-diffusion" regime along the biasing coordinate, i.e., the path progress parameter, s. This occurs before 2 ns for all sequences.
3. We calculate metadynamics-based free-energy profiles—i.e., the negative of the sum of Gaussian potentials—every 0.1 ns, starting at 2 ns and finishing at 7 ns for most sequences (See Supplementary Information for exceptions).
4. The mean of these free-energy estimations is taken as our final result, and their standard deviation as an error bar, which are reported in Fig. 2 and Fig. S2.

While metadynamics is our method of choice in this article, other techniques can also be used to sample along a path. Previously, we have reported similar free-energy profiles along WCF-to-HG paths obtained via US [23] and Constrained MD [39]. These profiles are consistent with our results for the CAA sequence.

In order to reproduce 1D free-energy profiles along s based on 2D free energy landscapes on the (χ',θ)-plane, such as the ones reported in [19] and [20], one requires to post-process the 2D data. Simply integrating the free energy along χ' or θ, while insightful and done in the two mentioned references, does not provide the same mechanistic optimization as a minimum free-energy path. In [41], a methodology is presented to optimize a string connecting two given states in a pre-calculated ND free-energy hypersurface. Such method (which our group has made available at: https://www.compchem.nl/software_package/trace-irc/) can be used to find MFEPs between the WCF and HG states, as well as the base-flipped intermediate state, on 2D free-energy landscapes calculated via metadynamics. With some adjustments, the method could also be employed on free-energy landscapes calculated via different methods.

Changes to the text:

- We have clarified the protocol to calculate the free-energy profiles and their error bars in Materials and methods.
- We have also added a note and reference on how to compare the 1D free-energy profiles along paths vs 2D free-energy surfaces in Conclusion.

2. In Figure 2B, the free energy difference between WC and HG in various sequences were shown. In principle, this free difference should be the same for the inside and outside paths. But non-negligible differences (up to several kcal/mol) were found in some sequences (CAC, CAG, ….). This may raise a concern on the reliability of this data. Also, in GAA, the free energy difference from the outside path (in green) has too much error. The authors should provide some reasonable explanations for these two issues.

We thank Reviewer #2 for this valuable comment. The maximum inconsistency for the free-energy difference between the inside and outside paths of the same sequence are indeed for CAC and CAG, with values of 1.9 and 2.3 kcal/mol respectively. Still, these—and all other sequences—have overlapping error bars. These disagreements are due to two factors: (1) the implicit error in the metadynamics estimation, (2) the tube potentials, which limit the sampling of the free-energy valleys in the perpendicular to the path, thereby modifying the distributions with respect to unrestrained sampling.

Concerning the error bar of the outside pathway of sequence GAA, and CAT, please see the answer of question 1 from Reviewer #1

Changes in the text:

- We added a comment about the consistency of the free-energy differences between inside and outside paths in Results and Discussion

3. For the outside paths, only the major groove opening path (negative bf angle) was considered. (In this paper, the bf angle (theta) is defined as negative for the major groove opening, while it was defined as positive in the original paper). Although they claim that the minor groove path is unlikely, previous papers on full 2D free energy maps (chi, theta) showed that the minor-groove path is quite possible. By matching the two CVs (chi', theta) used in this study, re-projection of the previously reported (chi, theta) to (chi', theta) map would not change the popularity of the minor groove path. The authors are invited to address this difference in discussion.

We thank Reviewer #2 for the attentive comment. Previous studies based on umbrella sampling (US) [19] and metadynamics combined with extended-system adaptive biasing force (meta-eABF) [20] have indeed showed 3 types of pathways: (1) without base flipping, (2) with base flipping toward the major groove, and (3) with base flipping toward the minor groove. In our work, we decided to focus only on the first two kinds after our previous transition path sampling (TPS) study [25] showed spontaneous flipping only towards the major groove in the CAA sequence. Given that the Monte Carlo sampling in trajectory-space done by TPS provides unbiased mechanisms, we left base flipping toward the minor groove out of the present study. However, and given that the current framework could in principle be extended to 3 pathways, we recognize the potential to address it in a future study.

Changes to the text:

- We have added the rationale behind our choice to focus on the inside and major groove pathways to Materials and methods and to Conclusion.

4. In Figure S1, in the 3' variation panel, there is a typo: CAG CAG ?CAT CAG?.

We thank Reviewer #2 for the correction.

Changes to the text:

- We have changed Figure S1 accordingly.

Reviewer #3: In this manuscript, the authors describe an enhanced sampling method, as applied to a base-flipping motion associated with the transition between canonical and Hoogsteen orientations. The primary conclusion is very intuitive: there is a smaller energetic barrier when the base first flips "out", rather than rearranging within the stacked bases of dsDNA. One certainly expects this result, since the sterics associated with base rotation in a confined environment will be extremely strong.

The manuscript is easy to read, and the work appears to be sound. However, the study is not suitable for PLOS Comp Bio. First, there is no clear connection to biology. That is, do the different barrier heights for different sequences explain a known biological phenomenon, or suggest new biological physics? As written, the results are primarily a display of the methodology. The second issue is that the general topic of enhanced sampling methods is not particular new. There are many methods available in the literature, and there is no comparison of the results with other methods. If this is a methods paper, then rigorous comparison should be provided. Comparisons would have to describe differences in accuracy and performance. This work would be more suitable for a simulation-methods-focused journal, such at JCTC.

We thank Reviewer #3 for the constructive criticism. The biological relevance of HG base pairing was recontextualized after the seminal NMR and simulation studies of Nikolova and coworkers [5, 7]. As we mention in the Introduction, HG populations can be as abundant as 0.5% in A-T and G-C base pairs, with residence times of up to 1.5 ms. The sequence composition has a definitive effect, with HG populations reaching up to 1.2% in A-T rich segments [6]. Moreover, within the several biological functions in which HG base pairing is involved together with DNA-protein complexes—e.g., replication, transcription and tumor suppression—there is also evidence of sequence dependence—e.g., in the TATA-box binding protein. Since HG and WCF base pairs coexist in a dynamical equilibrium, two important questions arise:

1. How does the sequence composition regulate the HG populations and residence times, i.e., the free energy difference and barrier from WCF to HG?

2. Does the sequence itself encode a particular preference for HG base pairing, or does is that enforced by the formation of a DNA-protein complex?

In our work, we set to answer the first question in a minimal system that is still comparable to previous computational results (note that almost all previous studies were done exclusively on the CAA sequence). As reported in the main text, we find that free-energy barriers for the inside pathway are affected by the direct neighbors, with G and C raising them. However, the outside pathway, which does not present such direct-neighbor dependence, is preferred across all sequences. We also find the lowest free-energy barrier and difference in the TAA sequence, agreeing with previous reports on the effect of A-T rich sequences. Most importantly, we find that the most dominant factor influencing HG proclivity is the direction of the rotation, which in our asymmetric chain is related to the relative flexibility toward either direction of the DNA segment. This gives some indication that DNA-binding proteins are related to stressed regions in DNA were HG base pairing is favored, as reported in [42]. Our results regarding (1) a preferred outside pathway, (2) a weak dependence on the direct neighbors, and (3) a strong flexibility-dependence, can guide future searches for HG base pairs in experimental structures, which might be mislabeled as WCF [42]. Recent publications have also posed that while WCF base pairs store static genetic information, HG base pairs can encode dynamic functions [43], which motivates efforts to understand their dynamical equilibrium.

Concerning the presentation of the methodology, details about the development and rigorous testing of path-based methods have been published previously in [22,23,29,31].

Changes to the text:

- We have added reference [42], corresponding to a recent study of HG base pairs in stressed protein-DNA complexes, and [43], corresponding to the functional importance of HG base pairs.
- We recapitulate the biological relevance described in the Introduction now also briefly in the Conclusion section to emphasize the impact of our findings.

Additional changes to the text not solicited by the reviewers:

- We have added a few pertinent references that we have found after the initial submission [31,45].
